# Rapid and Efficient CRISPR/Cas9-Based Mating-Type Switching of *Saccharomyces cerevisiae*

**DOI:** 10.1534/g3.117.300347

**Published:** 2017-11-17

**Authors:** Ze-Xiong Xie, Leslie A. Mitchell, Hui-Min Liu, Bing-Zhi Li, Duo Liu, Neta Agmon, Yi Wu, Xia Li, Xiao Zhou, Bo Li, Wen-Hai Xiao, Ming-Zhu Ding, Ying Wang, Ying-Jin Yuan, Jef D. Boeke

**Affiliations:** * Key Laboratory of Systems Bioengineering (Ministry of Education), School of Chemical Engineering and Technology, Tianjin University, 300072, China; † SynBio Research Platform, Collaborative Innovation Center of Chemical Science and Engineering (Tianjin), Tianjin University, 300072, China; ‡ Department of Biochemistry and Molecular Pharmacology NYU Langone Health, New York 10016; § Institute for Systems Genetics, NYU Langone Health, New York 10016

**Keywords:** *Saccharomyces cerevisiae*, mating-type switching, CRISPR/Cas9, ring chromosome, polyploidy

## Abstract

Rapid and highly efficient mating-type switching of *Saccharomyces cerevisiae* enables a wide variety of genetic manipulations, such as the construction of strains, for instance, isogenic haploid pairs of both mating-types, diploids and polyploids. We used the CRISPR/Cas9 system to generate a double-strand break at the *MAT* locus and, in a single cotransformation, both haploid and diploid cells were switched to the specified mating-type at ∼80% efficiency. The mating-type of strains carrying either rod or ring chromosome III were switched, including those lacking *HML*α and *HMR***a** cryptic mating loci. Furthermore, we transplanted the synthetic yeast chromosome V to build a haploid polysynthetic chromosome strain by using this method together with an endoreduplication intercross strategy. The CRISPR/Cas9 mating-type switching method will be useful in building the complete synthetic yeast (Sc2.0) genome. Importantly, it is a generally useful method to build polyploids of a defined genotype and generally expedites strain construction, for example, in the construction of fully **a/a**/α/α isogenic tetraploids.

The sex of *Saccharomyces cerevisiae* is determined by its mating-type locus, *MAT***a** or *MAT*α, typically manifesting as either a *MAT***a** or *MAT*α haploid or a *MAT***a**/α diploid. Strains of the opposite mating-type can mate to produce *MAT***a**/α diploids, which are incapable of mating (Hawthorne 1963). Mating-type switching is an important model for cell lineage, gene silencing, and genomic rearrangement analysis, and is also of great practical importance to construct an isogenic set (**a**, α, and **a**/α) of a given strain (Herskowitz and Jensen 1991; Haber 2012). Sc2.0 (the synthetic yeast genome project) aims to produce the first functional synthetic eukaryotic cell with redesigned chromosomes (Richardson *et al.* 2017). To efficiently consolidate all 16 individually synthesized chromosomes in a final functional cell, a number of sequential steps of mating-type switching and intercross will be needed. Thus, a rapid, simple, and highly efficient method of mating-type switching is critically needed to complete genome synthesis. More broadly, switching the mating-type of a strain is a standard but inefficient general method frequently used in strain construction, and enhancements in this method will be widely useful.

The *HO* gene (homothallic switching endonuclease gene) encodes a site-specific endonuclease that directs mating-type switching. The endonuclease generates a double-strand break (DSB) at the Z1 region of the *MAT* cassette in late G1 of the haploid cell cycle. Although the cleavage site is also encoded at the *HML*α and *HMR***a** loci, cleavage by *HO* endonuclease in the silent cassettes is prevented by the action of the *SIR* gene products (Strathern *et al.* 1982; Kostriken *et al.* 1983). Mating-type switching occurs by gene conversion of copies of silent mating-type cassettes from inactive loci, *HML*α and *HMR***a**, to the active mating-type locus, *MAT* (Rine and Herskowitz 1987; Haber 1998) (Figure 1A). Laboratory strains are mating-type stable and heterothallic by virtue of deletion or mutation of the *HO* gene. To isolate a strain of the opposite mating-type in the laboratory, a plasmid carrying the cloned *HO* gene can be transformed into the haploid strain to promote mating-type switching. The typical outcome of such an experiment is the subsequent mating within the population of interconverted and noninterconverted cells to yield isogenic *MAT***a**/α diploids. As such, to isolate the desired haploid cell of the opposite mating-type usually requires sporulation and tetrad dissection (Takahashi *et al.* 1958; Russell *et al.* 1986). *HO* gene expression can be placed under the control of a galactose promoter to allow for inducible mating-type interconversion (Herskowitz and Jensen 1991), although the efficiency of mating-type conversion is not reliably high (Nickoloff *et al.* 1986; Hou *et al.* 2013). Finally, if the *HML*α and *HMR***a** loci are deleted from a given strain, the mating-type cannot be switched by interconversion via expression of the *HO* gene (Klar *et al.* 1984). The published version of synthetic yeast chromosome III, synIII (Annaluru *et al.* 2014), lacks the silent cassettes; thus, every time it is consolidated with another synthetic chromosome it is necessary to switch the mating-type.

**Figure 1:**
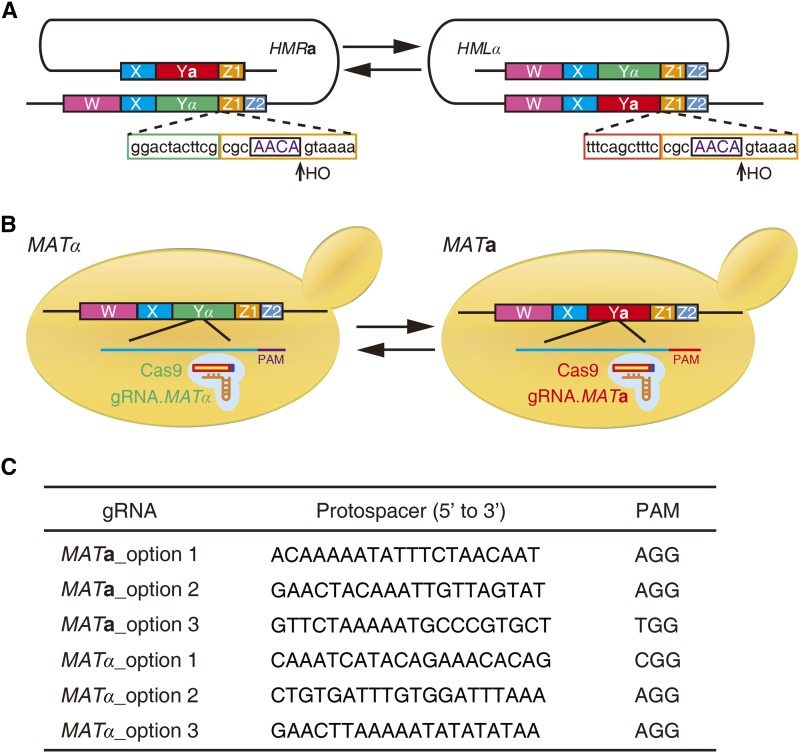
Schematic of the mating-type switching. (A) Mating-type interconversion between *MAT*a and *MAT*α by HO-induced DSB and templated HR. (B) Schematic outlining the CRISPR/Cas9-mediated mating-type switching. DSBs are generated by Cas9 specifically targeted to the Ya or Yα region. (C) Protospacer and PAM sequences for *MAT*a and *MAT*α mating-type switching.

Here we demonstrate a CRISPR/Cas9-mediated method to enable efficient mating-type switching in *S. cerevisiae*. We show isolation of wild-type haploid or diploid cells of the desired mating-type with high efficiency via a single yeast transformation of plasmids expressing Cas9 and optimal guide RNA (gRNA) sequences together with the appropriate donor *MAT* locus. We also successfully switch the mating-type of cells carrying synIII, which lack the *HML*α and *HMR***a** cryptic mating loci. Finally, we couple mating-type switching of a strain carrying synthetic chromosome V, synV (Xie *et al.* 2017), with an endoreduplication intercross to build a polysynthetic Sc2.0 strain encoding synV and synthetic chromosome X, synX (Wu *et al.* 2017). The CRISPR/Cas9 mating-type switching method is generally useful for construction of isogenic strains and polyploid strains of defined genotype.

## Materials and Methods

### Strains and plasmids

All *S. cerevisiae* strains used in this study are listed in Table 1. The strain yLM422 carries a synIII chromosome with mating-type α (Annaluru *et al.* 2014), and lacks *HML*α and *HMR***a**. The *MAT***a** derivative synIII strain yXZX621 and the *MAT*α derivative synV strain yXZX543 were constructed using the method described here.

**Table 1:** Yeast strains used in this study

Strain	Description	Parent	Source
*14***a**	*MAT***a** *his1*		Trueheart *et al.* (1987)
*17*α	*MAT*α *his1*		Trueheart (*et al.* 1987)
*BY4741*	*MAT***a** *his3*Δ*1 leu2*Δ*0 met15*Δ*0 ura3*Δ*0 LYS2*		Brachmann (*et al.* 1998)
*BY4742*	*MAT*α *his3*Δ*1 leu2*Δ*0 lys2*Δ*0 ura3*Δ*0 MET15*		Brachmann (*et al.* 1998)
*BY4743*	*MAT***a**/α *his3*Δ*1 leu2*Δ*0 lys2*Δ*0/LYS2 MET15/met15*Δ*0 ura3*Δ*0*		Brachmann (*et al.* 1998)
*yLM422*	*MAT*α *his3*Δ*1 leu2*Δ*0 lys2*Δ*0 ura3*Δ*0 MET15 synIII HO*::*synSUP61 hml*Δ *hmr*Δ		Annaluru (*et al.* 2014)
*yXZX621*	*MAT***a** *his3*Δ*1 leu2*Δ*0 lys2*Δ*0 ura3*Δ*0 MET15 synIII HO*::*synSUP61 hml*Δ *hmr*Δ	*yLM422*	This study
*yXZX716*	*MAT***a**/α *his3*Δ*1 leu2*Δ*0 lys2*Δ*0 ura3*Δ*0 MET15 synIII-272123bp/synIII-272123bp HO*::*synSUP61/HO*::*synSUP61*	*yLM422 x yXZX621*	This study
*yXZX880*	*MAT***a** *his3*Δ*1 leu2*Δ*0 met15*Δ*0 ura3*Δ*0 LYS2 ring_wtIII ring_wtIX*	*BY4741*	This study
*yXZX958*	*MAT*α *his3*Δ*1 leu2*Δ*0 met15*Δ*0 ura3*Δ*0 LYS2 ring_wtIII ring_wtIX*	*yXZX880*	This study
*yXZX959*	*MAT***a**/α *his3*Δ*1 leu2*Δ*0 met15*Δ*0 ura3*Δ*0 LYS2 ring_wtIII/ring_wtIII ring_wtIX/ring_wtIX*	*yXZX880 x yXZX958*	This study
*yXZX512*	*MAT***a** *his3*Δ*1 leu2*Δ*0 met15*Δ*0 ura3*Δ*0 LYS2 synV*		Xie (*et al.* 2017)
*yXZX543*	*MAT*α *his3*Δ*1 leu2*Δ*0 met15*Δ*0 ura3*Δ*0 LYS2 synV*		Xie (*et al.* 2017)
*yXZX547*	*MAT*α *his3*Δ*1 leu2*Δ*0 met15*Δ*0 ura3*Δ*0 LYS2 synV pGAL1-CEN10*::*Kl.URA3*	*yXZX543*	This study
*yYW0117*	*MAT***a** *his3*Δ*1 leu2*Δ*0 met15*Δ*0 ura3*Δ*0 LYS2 synX HO*::*tR(CCU)J*		Wu (*et al.* 2017)
*yYW0119*	*MAT***a** *his3*Δ*1 leu2*Δ*0 met15*Δ*0 ura3*Δ*0 LYS2 synX pGAL1-CEN5*::*Kl.URA3 HO*::*tR(CCU)J*	*yYW0117*	This study
*yXZX558*	*MAT***a**/α *his3*Δ*1 leu2*Δ*0 met15*Δ*0 ura3*Δ*0 LYS2 synV/wtV synX/wtX syn-CEN5/pGAL1-CEN5*::*Kl.URA3 syn-CEN10/pGA1L-CEN10*::*Kl.URA3 HO*::*tR(CCU)J/HO*	*yXZX547 x yYW0119*	This study
*yXZX572*	*MAT***a**/α *his3*Δ*1 leu2*Δ*0 met15*Δ*0 ura3*Δ*0 LYS2 synV synX HO*::*tR(CCU)J/HO*	*yXZX558*	This study
*yXZX573*	*MAT***a** *his3*Δ*1 leu2*Δ*0 met15*Δ*0 ura3*Δ*0 LYS2 synV synX HO*::*tR(CCU)J*	*yXZX572*	This study
*yXZX625*	*MAT*α *his3*Δ*1 leu2*Δ*0 met15*Δ*0 ura3*Δ*0 LYS2 synV synX HO*::*tR(CCU)J*	*yXZX573*	This study
*yXZX973*	*MAT***a**/**a** *his3*Δ*1 leu2*Δ*0 lys2*Δ*0/LYS2 MET15/met15*Δ*0 ura3*Δ*0*	*BY4743*	This study
*yXZX974*	*MAT*α/α *his3*Δ*1 leu2*Δ*0 lys2*Δ*0/LYS2 MET15/met15*Δ*0 ura3*Δ*0*	*BY4743*	This study
*yXZX975*	*MAT***a**/**a** *his3*Δ*1 leu2*Δ*0 lys2*Δ*0 ura3*Δ*0 MET15 synIII/synIII HO*::*synSUP61/HO*::*synSUP61*	*yXZX716*	This study
*yXZX976*	*MAT*α/α *his3*Δ*1 leu2*Δ*0 lys2*Δ*0 ura3*Δ*0 MET15 synIII/synIII HO*::*synSUP61/HO*::*synSUP61*	*yXZX716*	This study
*yXZX979*	*MAT***a**/**a**/α/α *his3*Δ*1 leu2*Δ*0 lys2*Δ*0/LYS2 MET15/met15*Δ*0 ura3*Δ*0*	*yXZX973 x yXZX974*	This study
*yXZX980*	*MAT***a**/**a**/α/α *his3*Δ*1 leu2*Δ*0 lys2*Δ*0 ura3*Δ*0 MET15 synIII/synIII/synIII/synIII HO*::*synSUP61/HO*::*synSUP61*	*yXZX975 x yXZX976*	This study

A plasmid encoding the Cas9 gene, pNA0306 (pRS415-TEF1p-Cas9), was modified from Addgene plasmid #43802. The plasmid expressing one gRNA, pNA0304 (pRS426-SNR52p-gRNA-SUP4t), was modified from Addgene plasmid #43803. The plasmid expressing two gRNAs simultaneously was pNA0308 (p426-SNR52p-gRNA-SUP4t-pRPR1-gRNA-RPR1t) (Table 2). Phusion DNA polymerase and restriction enzymes were purchased from New England Biolabs.

**Table 2:** Plasmids used in this study

Plasmid No.	Plasmid Name	Description	Yeast Marker	*Escherichia* Marker	RE*	Source	Addgene ID
	YCplac33-*GAL2p-HO-KanMX4*	*pGAL2-HO* plasmid	*KanMX4*, *URA3*	Amp	N/A	Hou (*et al.* 2013)	
	pRS426	Yeast episomal vector	*URA3*	Amp	N/A	Christianson (*et al.* 1992)	
pXZX010	pRS426-*GAL2p-HO-KanMX4*	*pGAL2-HO* plasmid	*KanMX4*, *URA3*	Amp	N/A	This study	
pNA0306	pRS415-*TEF1p*-Cas9-*CYC1t*	Cas9 expression plasmid	*LEU2*	Amp	N/A	DiCarlo (*et al.* 2013)	#43802
pNA0304	pRS426-*SNR52p*-gRNA-*SUP4t*	Backbone plasmid for gRNA insertion	*URA3*	Amp	N/A	DiCarlo (*et al.* 2013)	#43803
pNA0308	pRS426-*SNR52p*-gRNA-*SUP4t-RPR1p*-gRNA-*RPR1t*	Double gRNA backbone plasmid	*URA3*	Amp	N/A	This study	
pXZX339	pRS426-*SNR52p*-gRNA.*MAT***a_**option1-*SUP4t*	20-bp gRNA sequence targeting *MAT***a** cassette	*URA3*	Amp	N/A	This study	
pXZX388	pRS426-*SNR52p*-gRNA.*MAT***a_**option2-*SUP4t*	20-bp gRNA sequence targeting *MAT***a** cassette	*URA3*	Amp	N/A	This study	
pXZX501	pRS426-*SNR52p*-gRNA.*MAT***a_**option3-*SUP4t*	20-bp gRNA sequence targeting *MAT***a** cassette	*URA3*	Amp	N/A	This study	
pXZX351	pRS426-*SNR52p*-gRNA.*MAT*α_option1-*SUP4t*	20-bp gRNA sequence targeting *MAT*α cassette	*URA3*	Amp	N/A	This study	
pXZX503	pRS426-*SNR52p*-gRNA.*MAT*α_option2-*SUP4t*	20-bp gRNA sequence targeting *MAT*α cassette	*URA3*	Amp	N/A	This study	
pXZX504	pRS426-*SNR52p*-gRNA.*MAT*α_option3-*SUP4t*	20-bp gRNA sequences targeting *MAT*α cassette	*URA3*	Amp	N/A	This study	
pXZX352	pTOPO-*MAT***a**	*MAT***a** fragment in pTOPO vector	—	Kan	*Nsi*I	This study	
pXZX353	pTOPO-*MAT*α	*MAT*α fragment in pTOPO vector	—	Kan	*Nsi*I	This study	
pXZX448	pRS426-*SNR52p*-gRNA.proto_chr3_L-*SUP4t-RPR1p*-gRNA-*RPR1t*	20-bp gRNA sequence targeting left end of chromosome *III*	*URA3*	Amp	N/A	This study	
pXZX406	pRS426-*SNR52p*-gRNA.proto_chr3_L-*SUP4t-RPR1p*-gRNA.proto_chr3_R-*RPR1t*	20-bp gRNA sequences targeting both left and right ends of chromosome *III*	*URA3*	Amp	N/A	This study	
pLM182		*pGAL1-CEN5-Kl.URA3*	*Kl.URA3*	Amp	*Not* I	Reid (*et al.* 2008)	
pLM187		*pGAL1-CEN10-Kl.URA3*	*Kl.URA3*	Amp	*Not *I	Reid (*et al.* 2008)	

* RE, restriction enzyme

The *GAL2p-HO-KanMX4* cassette was polymerase chain reaction (PCR) amplified from plasmid YCplac33-GHK with primers oXZX011 and oXZX012, which had 40-bp overlaps identical to the ends of *EcoR*I-digested plasmid pRS426 (Hou *et al.* 2013; Lin *et al.* 2015). pXZX010 was assembled in yeast by cotransforming gel-purified *EcoR*I-digested pRS426 and the *GAL2p-HO-KanMX4* PCR amplicon, followed by selection on synthetic complete medium lacking uracil (SC–Ura) (Table 2 and Table 3).

**Table 3:** Primers used in this study

Number	Sequence
oXZX011	GCCCCCCCTCGAGGTCGACGGTATCGATAAGCTTGATATCTATTACATTTACTGAGCATAACGGGC
oXZX012	TGGCGGCCGCTCTAGAACTAGTGGATCCCCCGGGCTGCAGGCAGTATCGATCGACAGCAGT
oXZX292	ACGATAACTGGTTGGAAAGCGTAA
oXZX293	AGACTTGTGGCGAAGATGAATAGT
oXZX673	CTCACCCGAAAGAGATGCTG
oXZX674	GTTCTGTATAGTACATCCGCGCACATTCTTCCTAGCGGAAAGTCTTTACCGACGCTTGTG
oXZX675	TGTGTCGTACTTTTTATTTTCACAAGCGTCGGTAAAGACTTTCCGCTAGGAAGAATGTGC
oXZX676	TGCTATTGCCTATGTTCGCTC
oXZX677	TATGGACACAAACCATGCCTAACC
oXZX678	GCGCCTCCGAACAAACAGATG
oLM651	CGCCTCCGCAACTTCCTG
oLM178	TCAGCAATTTCGATGCAACCGG
oXZX356	TTTGTAGTTTGCGTCTTGTTCCG

### Construction of donor DNA containing plasmids

Oligonucleotides oXZX292 and oXZX293 were used to amplify a 3293-bp *MAT***a** cassette donor DNA fragment from BY4741 genomic DNA and a 3398-bp *MAT*α cassette donor DNA fragment from BY4742 genomic DNA (Table 3). The PCR products, after gel purification, were subcloned into a pCR-Blunt II-TOPO vector (45-0245; Invitrogen/Life Technologies, Carlsbad, CA) to generate the donor DNA carrying plasmids of pXZX352 (pTOPO-*MAT***a**) and pXZX353 (pTOPO-*MAT*α). These two plasmids can be transformed into the yeast for mating-type switching directly upon *Nsi*I digestion, which excises the inserts.

### Construction of gRNA expression plasmids

The 20-bp protospacers were designed in the Y**a** or Yα regions that precede the requisite protospacer-adjacent motif (PAM) to generate a specific DSB at *MAT***a** or *MAT*α (Figure 1B). The gRNA expression plasmid pNA0304 was digested by *Not*I and gel purified. Subsequently, the 20-bp gRNA cassettes of *MAT* locus, produced by annealing two single-strand oligonucleotides containing a 20-bp gRNA fragment and flanked by 20-bp overlaps identical to the backbone plasmid at the cut site, were assembled to the linearized backbone plasmid by using NEB Gibson Assembly Master Mix (E2611; New England BioLabs, Beverly, MA) (Figure 1C and Table 2) (Gibson 2011). All oligonucleotides were purchased from Integrated DNA Technologies (Coralville, IA) and Genewiz (Beijing, China).

Cleavage at the ends of chromosome III was generated by a double-gRNA expression plasmid, which was constructed by inserting two 20-bp protospacers into the double-gRNA backbone plasmid pNA0308 in two steps. First, the protospacer sequence “proto_chr3_L” was Gibson assembled into *Not*I-digested pNA0308 to obtain the intermediate plasmid pXZX448. Second, the protospacer sequence “proto_chr3_R” was Gibson assembled into the *Hind*III-digested pXZX448 to result in the double-gRNA expression plasmid pXZX406.

### Ring_wtIII chromosome construction

**Figure 2:**
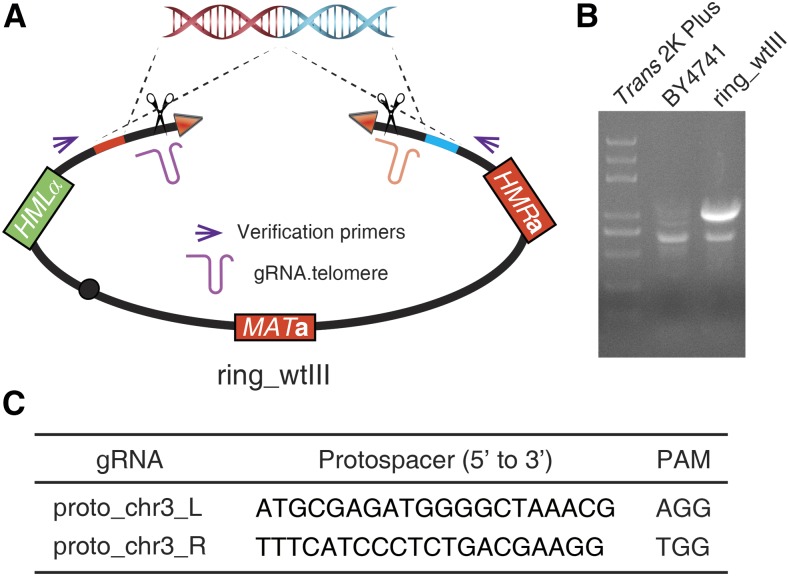
Construction and verification of ring_wtIII chromosome. (A) Circularization of wtIII yielded a designer version of ring_wtIII (yXZX880) without removal of *HML*α and *HMR*a. (B) PCR verification of ring_wtIII. (C) Protospacer and PAM sequences for wtIII cyclization. Note that the ring_wtIII structure constructed here is distinct in sequence from ring chromosomes isolated by classical methods by Haber *et al.* (1980).

A *MAT***a** ring chromosome III derivative, ring_wtIII (yXZX880), was cyclized using a homologous recombination (HR) strategy by deleting the telomere ends of native chromosome III, wtIII, and DSBs were generated by cleaving the two subtelomeric regions with CRISPR/Cas9 (Figure 2A and Table 1). Ring_wtIII encodes all genes present on parental wtIII, including *HML*α and *HMR***a**, except for deletions of 6275 bp on the left end and 1285 bp on the right end of wtIII, which was confirmed by the appearance of a PCR amplicon of oXZX677 and oXZX678, spanning the extreme chromosome termini (Figure 2, B and C and Table 3). The strain carrying *MAT*α ring_wtIII (yXZX958) was obtained by switching the mating-type of yXZX880 using the method described here (Table 1). Thus, this “designer” ring chromosome is distinct from previously isolated ring *III* chromosomes, which had many deleted genes (Haber *et al.* 1980).

The donor DNA fragment for chromosome III cyclization via HR was obtained by an overlapping-extension PCR strategy. First, the left homologous arm was PCR amplified from genomic DNA of BY4741 using primers oXZX673 and oXZX674, and the right homology arm was amplified from genomic DNA of BY4741 using primers oXZX675 and oXZX676. There was an 80-bp overlap between the two homologous arms. Second, left and right homologous fragments, after gel purification, were extended and amplified by using primers oXZX673 and oXZX676 (Table 3).

### Endoreduplication intercross

As described elsewhere, the “endoreduplication intercross” strategy is a powerful means to consolidate chromosomes and build polysynthetic yeast strains (Mitchell *et al.* 2017; Richardson *et al.* 2017). In such a cross, native chromosomes are modified by inserting a *GAL1* promoter, capable of destabilizing an adjacent centromere, together with a *URA3* gene, which can be selected against using 5-fluoroorotic acid (5-FOA). After the strains are crossed, induction of the *GAL1* promoter leads to destabilization of the native chromosomes and yields hemizygotes for the corresponding synthetic chromosomes, which are capable of endoreduplication to generate a 2n state and can be sporulated. We utilized the rapid mating-type switching method described here combined with an endoreduplication intercross to consolidate synV and synX in a single cell.

First, haploid yeast strains with conditional centromeres were constructed by integrating the CEN-conditional DNA segment, gel-purified *Not*I-digested pLM182 (pGAL1-CEN5) or pLM187 (pGAL1-CEN10) (Reid *et al.* 2008), into the corresponding native chromosome. The integration of CEN-conditional V was identified by primers oLM651 and oLM178, and the integration of CEN-conditional X was identified by primers oXZX356 and oLM178. Second, the synV strain carrying CEN-conditional X and the synX strain carrying CEN-conditional V were mated to generate a heterozygous diploid synthetic strain (synV/wtV synX/wtX), which was induced with galactose to destabilize the conditional native chromosomes. Third, the loss of CEN-conditional chromosomes was selected on 5-FOA medium and confirmed with PCRTag analysis. Fourth, sporulation and dissection were carried out to build the haploid *MAT***a** synthetic strain (synV/synX, yXZX573). Subsequently, the derived *MAT*α synV/synX strain yXZX625 was produced using the method described here.

### Yeast transformation of plasmids with donor DNA

Before transformation, 1 µg of *MAT* cassette carrying plasmid (pTOPO-*MAT***a** or pTOPO-*MAT*α) was digested with *Nsi*I in a final volume of 10 μl, and the whole digestion product was used directly for yeast transformation along with ∼200 ng of Cas9 expression plasmid (pNA0306) and ∼200 ng of gRNA expression plasmid (pXZX352 or pXZX353). Yeast transformation was carried out using a modified lithium acetate transformation method as described elsewhere (Mitchell *et al.* 2015).

To switch *MAT***a** to *MAT*α, the digestion product of pXZX353, gRNA expression plasmid pRS426-SNR52p-gRNA.*MAT***a**-SUP4t and Cas9 expression plasmid pNA0306 were cotransformed, while the digestion product of pXZX352, gRNA expression plasmid pRS426-SNR52p-gRNA.*MAT*α-SUP4t and Cas9 expression plasmid pNA0306 were cotransformed to switch *MAT*α to *MAT***a**. For each transformation, following a primary selection on SC–Ura–Leu plates for 2 d at 30°, transformants with the opposite mating-type were verified by replica plating to lawns of the tester strains.

During evaluation of mating-type switching efficiency, we first transformed the Cas9 expression plasmid into the strain and subsequently transformed the gRNA expression plasmid and donor *MAT* locus into the Cas9-expressing cells.

### Mating-type switching by pRS426-GHK

Galactose-induced *HO* plasmid pXZX010 (pRS426-*GAL2p-HO-KanMX4*) was used to test the mating-type switching efficiency by modifying the described method (Herskowitz and Jensen 1991). One single colony of pXZX010 was picked from a SC–Ura plate and inoculated into 3 ml of SC–Ura medium at 30° for 24 hr. Subsequently, cells were harvested and washed three times with sterile water, followed by inoculation in 3 ml SC–Ura + 2% glycerol medium dextrose glycerol overnight at 30°. The pellets were spun down, washed three times with sterile water, and resuspended in 3 ml SC–Ura 2% galactose medium to an A_600_ = 0.6–1.0 for 5 hr at 30°. A total of 200 µl of culture was removed after growth for 2, 3, 4, and 5 hr and samples were stored on ice. Aliquots were combined after 5 hr. After washing with sterile water, cultures were diluted and plated on yeast extract peptone dextrose (YPD) to obtain 100–200 colonies per plate and grown at 30° for 2 d.

### Mating-type test

Yeast mating-type determination was carried out by crossing the transformants with tester strains 17**a** and 14α, both carrying the *his1* auxotrophic marker. Lawns of tester strains were prepared by spreading ∼500 μl overnight YPD culture of tester strains on YPD agar plates and incubating at 30° for 2 d. The lawns and transformant colony plates were replica plated onto a fresh YPD plate at the same time. After incubating at 30° overnight, a second replica plating onto synthetic dextrose (SD) medium was performed and the plates were incubated at 30° to reflect mating response and scored the next day. Haploid strains and *MAT***a**/**a** or *MAT*α/α diploid strains should form a patch on just one of the two tester strains if the tester strain is of the opposite mating-type. *MAT***a**/α strains are nonmaters and will not form a prototrophic patch with either tester strain. Transformants able to mate with both an **a** lawn and an α lawn were scored as “bimater”.

### Plasmid loss

A single colony with verified mating-type was inoculated into 5 ml of YPD medium at 30° overnight to saturation. Then, 100 μl of cultures with 10^−5^ dilutions were plated on a SC + 5-FOA plate and incubated at 30° for 2 d, following replica plating onto a SC–Leu plate. The plasmid-free colonies can grow on SC + 5-FOA plates but not on SC–Leu plates.

### Data availability

The authors state that all data necessary for confirming the conclusions presented in the article are represented fully within the article.

## Results and Discussion

### Cas9-gRNA expression leads to highly efficient mating-type switching

**Figure 3:**
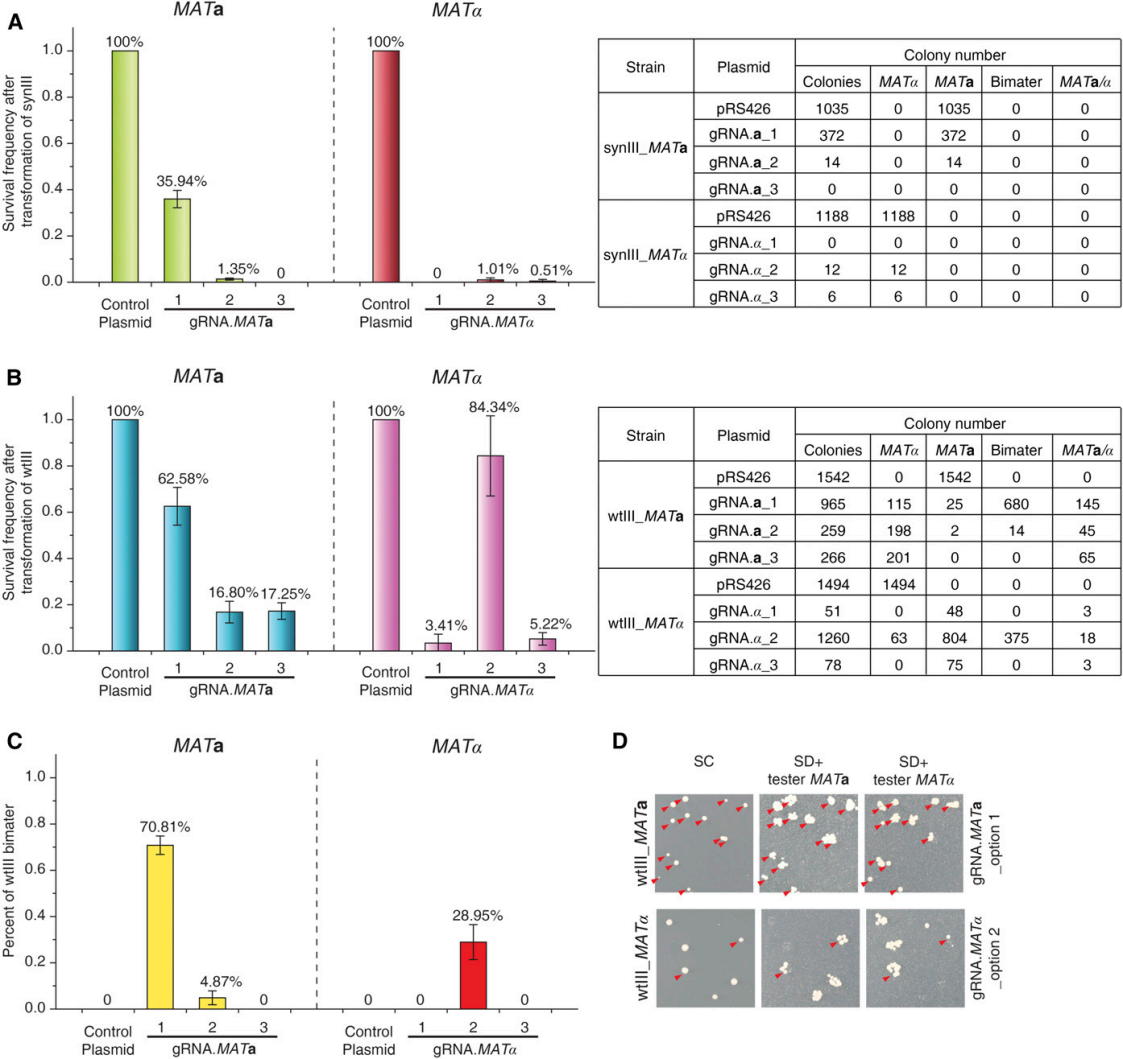
Selection of optimal gRNAs for efficient mating-type switching. (A) Left bar graph shows survival frequency of synIII strains (yXZX621 and yLM422) after transformation of gRNA plasmids without donor DNA. Table indicates analysis of colony types. *MAT*a/α genotype is deduced from nonmater phenotype. Survival frequency is defined as the percentage of surviving colonies recovered following transformation of a gRNA relative to an empty plasmid. (B) Same as (A) but with wtIII strains (BY4741 and BY4742). (C) Percentage of wtIII bimaters after transformation of gRNA plasmid without donor DNA. (D) Bimating phenotype of CRISPR/Cas9-mediated mating-type switching. Red triangle indicates bimater. Control plasmid is pRS426. Values are averages from three experiments, and error bars denote standard deviation SC, synthetic complete medium.

When a DSB is introduced at *MAT* in cells lacking the donor sequences *HML*α and *HMR***a**, for instance, by endogenous HO endonuclease or by CRISPR/Cas9, in principle all cells should die because the DSB cannot be repaired by HR (Klar *et al.* 1984). SynIII strains lack the silent cassettes (Annaluru *et al.* 2014) such that cleavage at *MAT* cannot be repaired by *HML*α or *HMR***a**, and mating-type switching absolutely requires a donor molecule. An optimal gRNA to direct mating-type switching should generate a DSB at *MAT* that promotes HR with minimal off-target effects. A strain carrying synIII represents the ideal genetic background to identify optimal gRNAs, since following formation of a DSB all cells should die; the surviving colonies could result from mutant sites refractive to DSB or perhaps off-target HR effects. To identify an optimal gRNA for mating-type switching, in synIII cells we tested three independent gRNA sequences that directed Cas9-mediated breaks to different positions within *MAT***a** or *MAT*α, specifically within the Y**a** or Yα regions (Figure 1, B and C). We counted the number of surviving colonies relative to a strain transformed with a plasmid lacking the gRNA, reporting a “survival frequency.” We also tested the mating-type of surviving colonies by replica-plating, using specific tester strains of known mating-type and carrying a specific auxotrophic marker complemented by mating (Trueheart *et al.* 1987). We observed a very low survival frequency of ∼2% following transformation of gRNA.*MAT***a**_option 2 or 3 into *MAT***a** derivative synIII cells. On the other hand, we observed a nearly 30% survival frequency for gRNA.*MAT***a**_option 1. The mating-type of the surviving cells for all three gRNAs was **a**. In the synIII strain encoding *MAT*α and using gRNA.*MAT*α_option 2 or 3, we recovered ∼1% the number of colonies relative to an empty vector lacking gRNA. For gRNA.*MAT*α_option 1, no colonies survived. The mating-type of the surviving colonies was all α (Figure 3A).

We also carried out this experiment in a wtIII strain, expecting that gene conversion templated by *HML*α or *HMR***a** would yield viable colonies and that the survival frequency would be high. Notably, after *MAT* locus conversion, the PAM sequence targeted by either gRNA is absent, preventing ongoing Cas9 cleavage at *MAT*. We counted the number of surviving colonies and evaluated their mating type. In the wtIII strain of *MAT***a** and using gRNA.*MAT***a**_option 2 or 3, we observed a survivor frequency of 17%. Interestingly, we recovered a relatively high survival frequency in the *MAT***a** derivative wtIII strain using gRNA.*MAT***a**_option 1 (62.58%). Among the surviving cells of gRNA.*MAT***a**_option 1, ∼70% were bimaters and only ∼12% were switched to α. On the other hand, for gRNA.*MAT*α_option 1 or 3, frequency of colonies was ∼5% relative to an empty vector lacking gRNA. The mating-type of these colonies was all α. In the wtIII strain encoding *MAT*α, we recovered ∼84% the number of colonies relative to an empty vector lacking gRNA, after transformation of gRNA.*MAT*α_option 2, in which ∼29% of surviving colonies were bimaters and ∼64% were switched to *MAT***a** (Figure 3, B–D).

In summary, based on the best combination of low survival frequency in the synIII background, lack of bimaters in a wtIII background, and a high switching efficiency, we selected gRNA.*MAT***a**_option 3 for mating-type switching from **a** to α, and gRNA.*MAT*α_option 1 for switching from α to **a** for all future work. The nature of the bimater phenotype observed in the wild-type background with less effective gRNAs is not clear to us at this time, but we note that (a) this phenomenon only occurs in the presence of silent cassettes and thus may result from low-level cutting at the *HM* loci, and (b) bimater formation is correlated with high levels of survival, suggesting relatively inefficient cutting by these gRNAs. By focusing on the two above optimal gRNAs, this class of transformants is minimized.

### Enhancing mating-type switching efficiency of haploid strains

**Figure 4:**
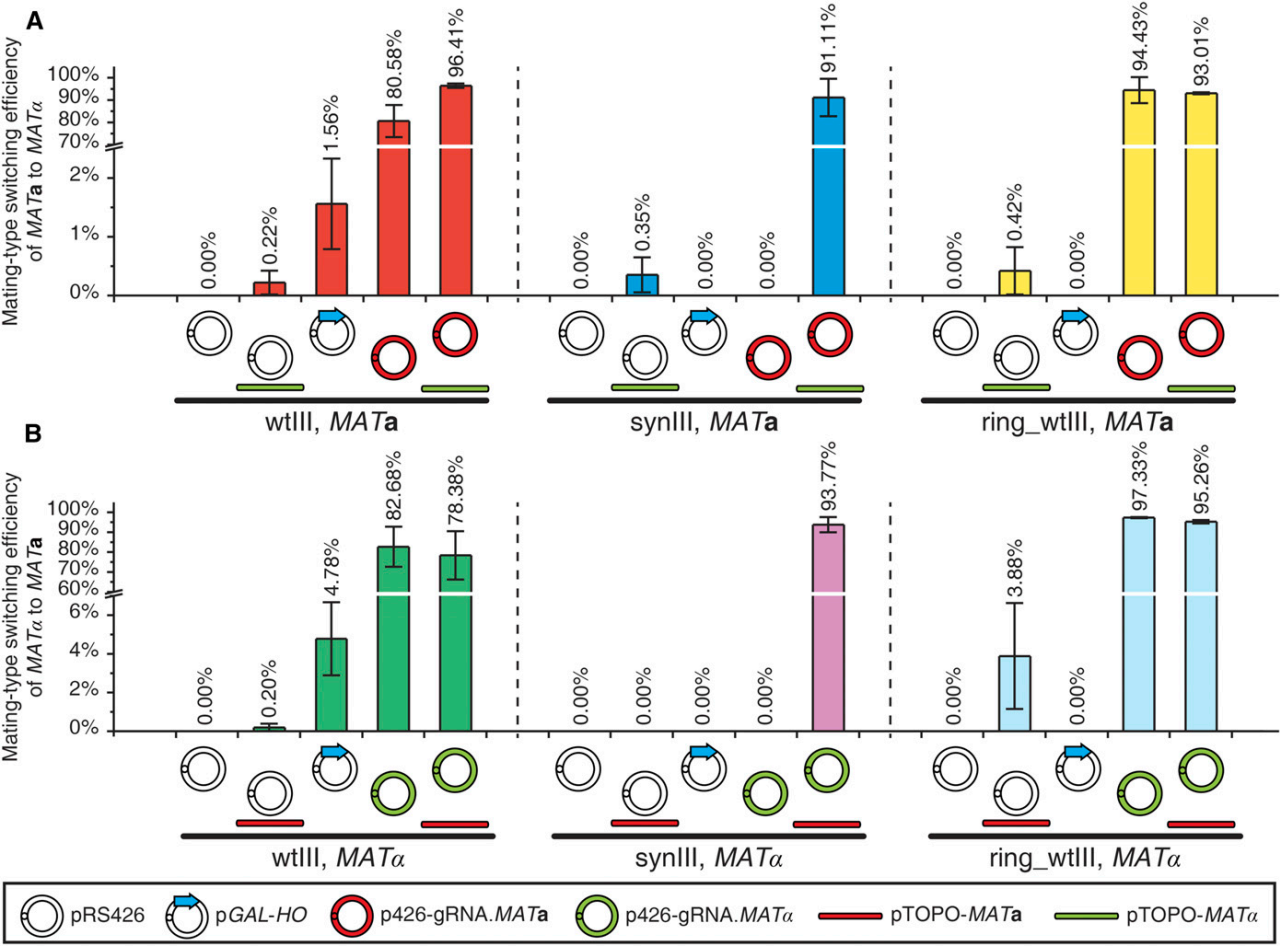
Efficiency of CRISPR/Cas9-mediated mating-type switching. (A) Mating-type switching efficiency of haploid strains from *MAT*a to *MAT*α. (B) Mating-type switching efficiency of haploid strains from *MAT*α to *MAT*a. Error bars are as in Figure 3.

To evaluate mating-type switching efficiency in haploids using the optimal gRNAs, three different types of haploid strains were selected, wtIII, synIII, and ring_wtIII. As described before, haploid strains carrying synIII lack *HML*α and *HMR***a** cassettes, and no transformants with the opposite mating-type are recovered in the absence of a cotransformed donor DNA segment. Thus, the donor DNA segment is essential for mating-type switching in synIII strains. When the donor DNA and gRNA plasmid were cotransformed into synIII strains, ∼91% of the surviving colonies were switched to the mating-type of interest regardless of the initial mating type, whereas only a small percentage of switched colonies were recovered by inducing the *GAL2p-HO* plasmid. Notably, mating-type switched colonies via the *GAL2p-HO* system were recovered about five times more efficiently when switching from α to **a** than from **a** to α, a further practical limitation of the GAL-based system. For the haploid strains carrying wtIII and ring_wtIII, each encoding *HML*α and *HMR***a**, ∼80–98% of viable colonies were switched in the absence of donor DNA, providing gRNA and Cas9 expression plasmid only. To test whether providing donor DNA for HR would improve the mating-type switching efficiency in strains encoding the *HML*α and *HMR***a** cryptic mating loci, we cotransformed donor DNA with Cas9 and gRNA to strains carrying wtIII or ring_wtIII; no significant improvement was observed (Figure 4, A and B).

### Building the haploid yeast with polysynthetic chromosomes

**Figure 5:**
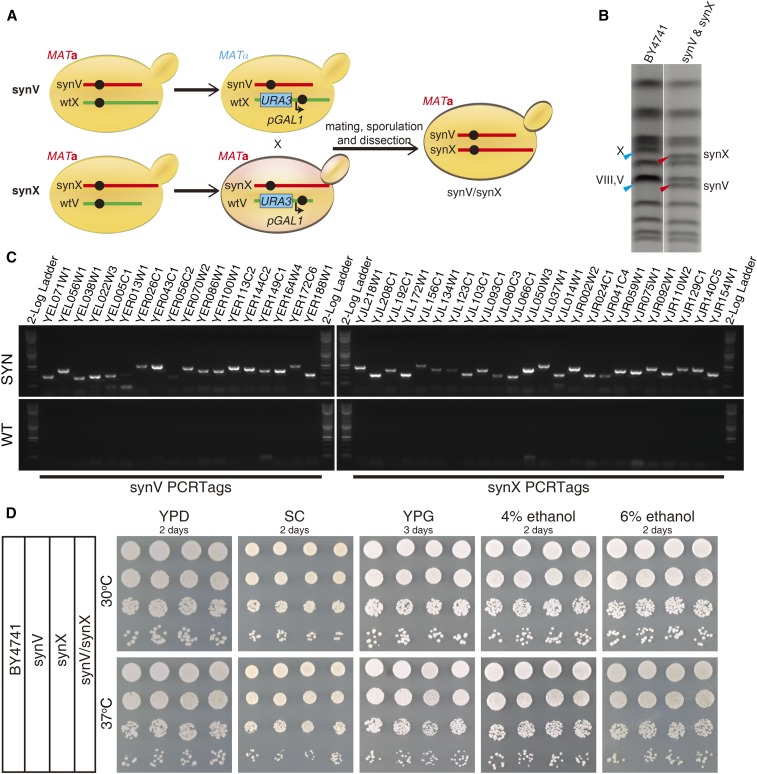
Construction and characterization of polysynthetic haploid strain synV/synX. (A) The mating-type of the synV strain was switched to *MAT*α with the CRISPR/Cas9-mediated strategy. Next, the *pGAL1-CEN10-Kl.URA3* construct was integrated into the wtX chromosome of synV strain, the *pGAL1-CEN5-Kl.URA3* construct was integrated into the wtV chromosome of synX strain, and the two strains were mated. The diploid strain was induced in galactose to destabilize the native chromosomes and confirmed by selection on FOA medium followed by PCRTag analysis. The final haploid polysynthetic chromosome strain synV/synX was generated by sporulation and dissection. Red indicates synthetic chromosome and green indicates native chromosome. (B) PFGE of synV and synX chromosomes. Red triangle indicates synthetic chromosome and blue triangle indicates wild-type chromosome. (C) PCRTag analysis (one PCRTag per ∼30 kb) of the polysynthetic haploid strain synV/synX. (D) Phenotypic analysis of synV, synX, synV/synX, and native (BY4741) strains under different conditions. SC, synthetic complete medium; SYN, synthetic PCRTags; YPD, yeast extract peptone dextrose; YPG, yeast extract peptone glycerol; WT, wild-type PCRTags.

synV and synX are two of the large synthetic yeast chromosomes, with a total length of ∼1.2 Mb, corresponding to 10% of the Sc2.0 genome (Richardson *et al.* 2017; Wu *et al.* 2017; Xie *et al.* 2017). We transplanted and consolidated the two chromosomes into a haploid polysynthetic strain, synV/synX (yXZX573), by coupling the CRISPR/Cas9-mediated mating-type switching method with the endoreduplication intercross strategy (Figure 5A). *CEN*-conditional V and *CEN*-conditional X native chromosomes were successfully destabilized to generate a 2n − 2 diploid.

No significant sporulation defect was observed in the synV/synX diploid, suggesting that the combination of the two synthetic chromosomes was sufficient to direct meiosis. Both four viable spore and two viable spore tetrads were observed. The four viable spores were consistent with the endoreduplication of two synthetic chromosomes prior to sporulation, and the two viable spore tetrads suggested the two synthetic chromosomes had not yet endoreduplicated (Mitchell *et al.* 2017; Richardson *et al.* 2017). Since *tR*(*CCU*)*J*, a single copy tRNA gene, was relocated from synX to the *HO* locus on chromosome IV in the synX strain (Wu *et al.* 2017), the expected ratio between the slow growth (sick) phenotype and normal growth phenotype is 2:2 in the four viable spore tetrads. On average it was 1:1 in the two viable spore tetrads; both results are as expected.

The size alterations of synV and synX were demonstrated by means of pulsed-field gel electrophoresis (PFGE) (Figure 5B). The genotype of synV/synX was characterized by PCR using both synthetic (SYN) and wild-type (WT) PCRTag primer pairs. The presence of SYN amplicons and absence of WT amplicons revealed the successful isolation of the polysynthetic haploid strain (Figure 5C). To evaluate the fitness of the polysynthetic strain, we examined the phenotype of synV, synX, synV/synX and the native strain (BY4741) under various conditions. No detectable differences were found in growth between the polysynthetic haploid strain and the native strain under most conditions (Figure 5D). The *MAT*α derivative synV/synX strain yXZX625 was produced using the method described here.

### Mating-type switching of diploids and construction of tetraploids

Polyploid and aneuploid strains are widely used in industrial manufacturing of chemicals, biofuels, and vaccines (Bidenne *et al.* 1992; Pfau and Amon 2012). To provide an efficient strategy to make polyploid strains of defined genotype, we evaluated mating-type switching efficiency in diploids and used CRISPR-Cas9 to construct tetraploids.

**Figure 6:**
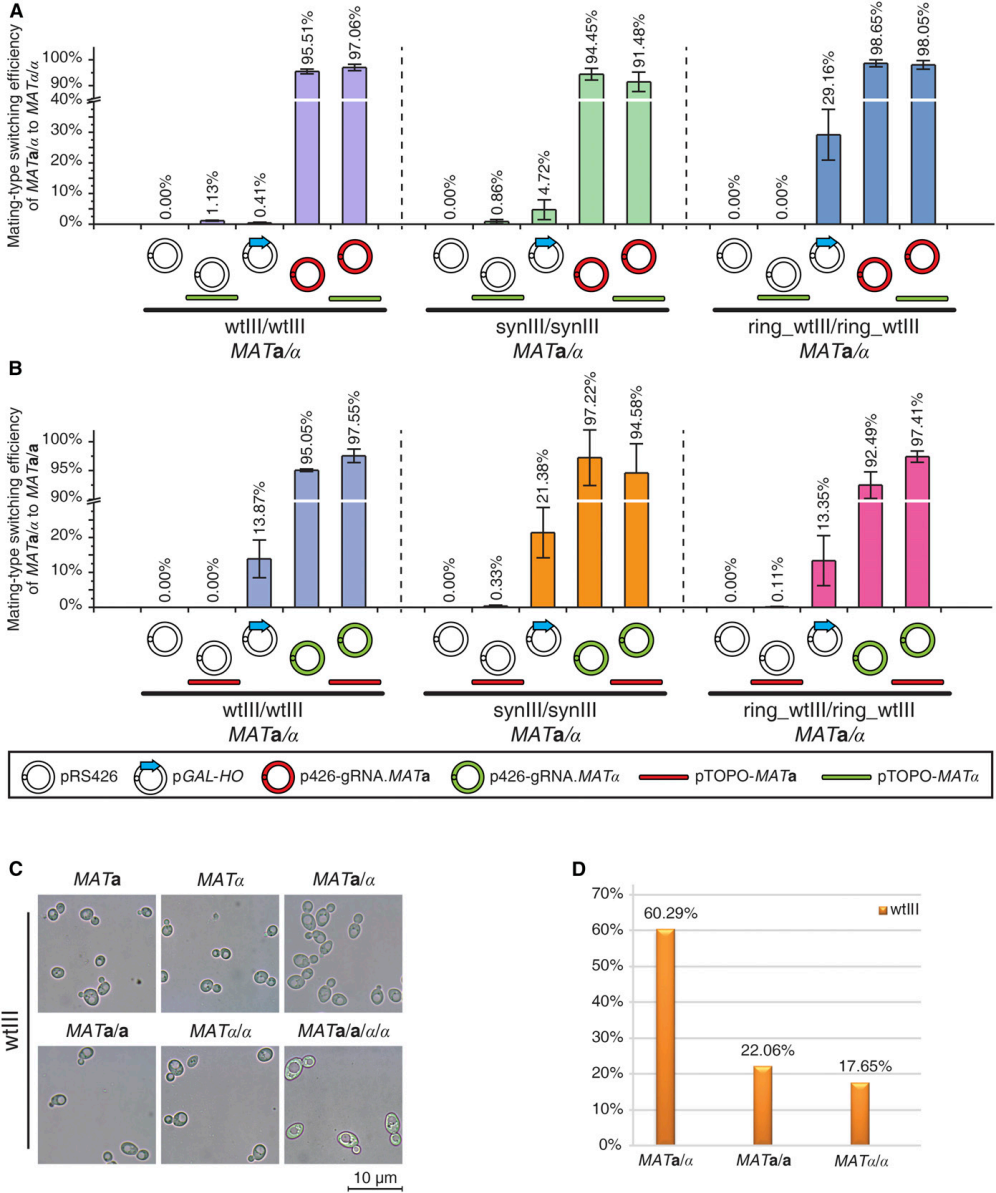
Mating-type switching of diploids and construction of tetraploid strains. (A) Mating-type switching efficiency of diploid strains from *MAT*a/α to *MAT*α/α. (B) Mating-type switching efficiency of diploid strains from *MAT*a/α to *MAT*a/a. (C) Morphology analysis of haploid, diploid, and tetraploid strains constructed with the method described here. (D) Tetrad analysis of tetraploids. The results are from 17 tetrads with four viable spores. Error bars are as in Figure 3.

Since the two gRNAs, gRNA.*MAT***a** and gRNA.*MAT*α, specifically target the *MAT***a** or *MAT*α locus, respectively, the CRISPR/Cas9-mediated mating-type switching method should work even in *MAT***a**/α diploid strains, a situation in which native *HO* is inactive. We attempted to switch the mating-type of *MAT***a**/α diploid strains to *MAT***a**/**a** and *MAT*α/α and measure switching efficiency in three kinds of *MAT***a**/α strains: wtIII*/*wtIII, synIII*/*synIII, and ring_wtIII/ring_ wtIII. Since the *MAT* locus on the second homolog serves as a donor for HR, >90% of surviving cells were switched to *MAT***a**/**a** or *MAT*α/α, without transforming donor DNA. Similar to the case of haploid strains, donor DNA cotransformation did not improve the mating-type efficiency notably (Figure 6, A and B).

Mating of diploids with *MAT***a**/**a** and *MAT*α/α produced isogenic tetraploids (yXZX979 and yXZX980); as expected, these demonstrated clearly larger cell size than the parental diploids (Figure 6C). After sporulation, we dissected 20 tetrads from yXZX979, and 17 of them produced four viable spores. When the mating-types of the resulting spores from the 17 tetrads was tested, 60% were nonmaters, 22% showed a mating-type **a**, and 18% showed a mating-type α, consistent with expectations (Figure 6D). The nonmating spores were inferred to be *MAT***a**/α, because they were capable of sporulation and produced tetrads with two *MAT***a** and two *MAT*α spores.

### Conclusions

Given the site-specificity and high efficiency of the CRISPR/Cas9 system, we describe a rapid, time-saving and highly efficient method to switch the mating-type of *S. cerevisiae* that is independent of both *HML*α/*HMR***a** and *HO*. We evaluated the gRNAs by minimizing the bimating phenotype and used this and the survival frequency to identify optimal gRNAs for this purpose. By using the CRISPR/Cas9-mediated mating-type switching method, a haploid strain of the opposite mating-type was isolated after a single cotransformation without the need for induction of *HO* expression, or downstream sporulation and tetrad dissection, although an isogenic diploid strain *MAT***a**/α may also be isolated directly. We constructed a polysynthetic haploid strain carrying synV and synX chromosomes, and built an isogenic set of strains carrying synIII, synV, and synV/synX using the method described here. This method will help promote completion of the synthetic genome of *S. cerevisiae*. Finally, *MAT***a**/α diploid strains were also switched to *MAT***a***/***a** and *MAT*α*/*α diploids with this method, and were further used to construct isogenic tetraploids. By repeating this process, construction of an isogenic polyploid series is possible.
